# Strengthening global health education in the UAE: assessing the current landscape and future directions

**DOI:** 10.3389/fpubh.2025.1584880

**Published:** 2025-06-12

**Authors:** Ali Artaman, Hillani T. Bekele, Ross Millar

**Affiliations:** ^1^Health Sciences Department, Zayed University, Dubai, United Arab Emirates; ^2^Department of Public Health and Epidemiology, Khalifa University, Abu Dhabi, United Arab Emirates; ^3^Health Services Management Centre, University of Birmingham, Birmingham, United Kingdom

**Keywords:** global healh, United Arab Emirates, education, capacity building, knowledge exchange

## Abstract

Global health is a growing area of interest for the United Arab Emirates (UAE). Given its location in the Middle East and its geographical proximity to developing sub-Saharan countries, the country is in a strategic position to contribute to global health agendas. Yet despite an increasing emphasis on global health policy, the availability of global health education programs at UAE universities remains limited. The aim of this Perspective is to raise awareness and understanding about the importance of the issue. Reflecting on the current landscape, it argues that it is crucial for higher education institutions in the UAE to better integrate global health into their curricula. The Perspective calls for a greater focus on curricula design, along with a further emphasis on collaboration and capacity building between universities and global health institutions to further strengthen this educational foundation.

## Introduction

Global health is a discipline that strives to transcend national borders (1). It is a perspective that seeks to go beyond the biomedical by engaging with the social, political, and economic determinants of health (2). Since the single-handed efforts of a nation can barely be successful in reducing the global burden of disease, multinational collaboration is essential to achieving a vision for global health (3).

Given its location in the Middle East and its geographical proximity to developing sub-Saharan countries, the United Arab Emirates (UAE) is in a strategic position to contribute to global health agendas. Global health is a key priority for the UAE (4), with the country maintaining and advocating for multilateral partnership to support universal health coverage and support the eradication of neglected tropical disease (4). The country continues to advocate for cooperation on issues like antimicrobial resistance, tuberculosis, and vaccine research (5).

Despite the increasing emphasis on global health issues, the availability of global health education programs at UAE universities remains limited. The aim of this Perspective is to raise awareness and understanding about the importance of health education for a future UAE global health policy. It argues that it is crucial for higher education institutions in the UAE to integrate global health into their curricula and calls for greater diversity of global health courses. To do so, the Perspective calls for greater collaboration and capacity building between universities and global health institutions to further strengthen this educational foundation.

### Background: global health and the UAE

Global health challenges such as noncommunicable diseases (NCD), workforce recruitment and retention, and climate change are of high significance to the UAE. Population health trends show that 77 per cent of all deaths in the UAE are attributed to noncommunicable diseases (NCD) such as cardiovascular disease, cancer, diabetes, and chronic respiratory diseases (6). The UAE healthcare system relies heavily on an expatriate health workforce and shortages are well documented due to the high turnover rate of workers who often leave the country after a few years (7). Attention is being paid to climate change and emergency preparedness, resilience and response where recent floods and environmental damage highlight the threats of climate change to the UAE and the wider region (8).

Actions are being undertaken within the UAE to respond to these various global health challenges. To support a global commitment to a 25 per cent reduction in premature NCD mortality by 2025, the UAE established a national action plan and the National Multisectoral NCD Committee (6). The UAE has also established The Global Institute for Disease Elimination (GLIDE) in 2017 through a partnership between His Highness Sheikh Mohamed bin Zayed Al Nahyan, president of the UAE, and the Bill & Melinda Gates Foundation (9). The UAE was the first country in the Middle East to sign and ratify the Paris Agreement and commit to an economy-wide reduction in emissions and a target of net zero by 2050. COP28, held in Dubai in 2023, signalled how the UAE would act as a global leader in climate change agendas and agreements.

These are all noble efforts, however, within these various responses to global health challenges are what we see as a missing link: the role of education. UAE policies such as The National Strategy for Higher Education 2030 are acknowledging the significance of education in the drive to economic development. The D33 Dubai Economic Agenda has reinforced this approach, highlighting how higher education sector growth is a crucial strategic priority for the coming decade (10). The UAE is in a critical position to educate and train the future health workforce. However, up to this point we argue that considerations of how global health policy and education agendas connect within the UAE have not received the attention they might have. Given the strategic importance and potential the UAE has in global health policy agendas, there is a need to engage with and promote global health education as foundation to achieving wider policy goals.

### Global health education in the UAE: mapping the landscape

Global health education is crucial to address current and future global health challenges. It can be an influential tool for achieving health equity, reducing health disparities, and developing professional careers (11). It not only equips students with the knowledge and skills that are essential to tackle transnational health issues, but also exposes learners to a range of successful global health solutions in other countries for similar problems.

**Figure 1:**
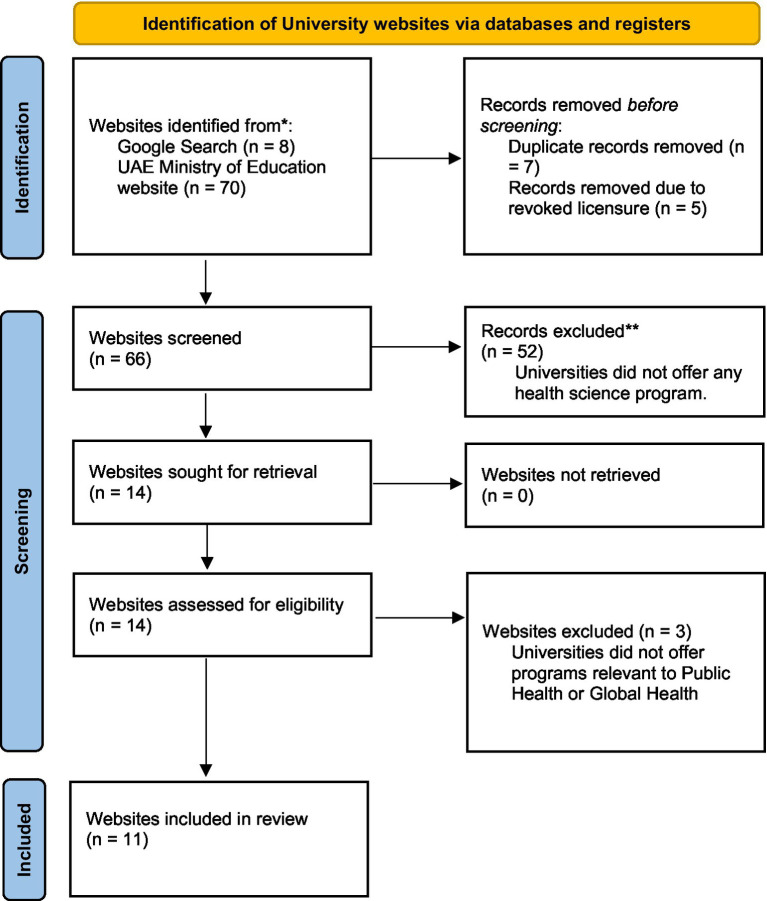
An outline of a “Global Health” program review process. Adapted from: Page et al. (18).

To get a better understanding of Global Health education offered in the UAE, we conducted a Google search using the terms “Global Health,” AND “Education OR Course,” AND “United Arab Emirates” (See Figure 1). This search was supplemented by a review of all licensed educational institutions in the UAE, as listed by the Ministry of Education. All institutions included were verified through a website of the UAE Ministry of Education (12).

Based on the inclusion criteria (Figure 1), we identified eleven universities and systematically examined the programs offered to determine whether a Global Health program was offered. Additionally, we reviewed course catalogs from relevant programs to identify whether any courses explicitly titled “Global Health” were offered.

### Inclusion and exclusion criteria

To identify the availability of Global Health courses and programs in the United Arab Emirates (UAE), we applied the following inclusion and exclusion criteria (Figure 1):

#### Inclusion criteria

- Institutions licensed by the UAE Ministry of Education were eligible for inclusion.
- Institutions were included if they offered at least one of the following academic programs
- Undergraduate or graduate Public Health programs (e.g., BSc, MSc, MPH, PhD).
- Programs in closely related disciplines, such as Health Administration, Community Health, or Health Promotion.
- Institutions offering any Health Sciences program (e.g., Medicine, Nursing, Pharmacy) were reviewed only if the course catalog explicitly listed a course or track titled “Global Health” or closely related topics (e.g., Global and International Health).

#### Exclusion criteria

- Institutions not recognized by the UAE Ministry of Education.
- Institutions that offered only health programs unrelated to public health (e.g., nursing, physiotherapy, veterinary medicine) and did not offer any course related to global health.

### Search timeframe

- The search was conducted from December 2024 to April 2025

### Criteria for classifying a course as “Global Health”

- Courses titled explicitly as “Global Health,” “Global and International Health,” or “Introduction to Global Health” were included.
- Courses on adjacent themes (e.g., Maternal and Child Health, Health Determinants) were not classified as Global Health.

### Language restriction

- The search included institutions with Arabic or English websites and no language restrictions were applied as long as the content was accessible and verifiable.

Our analysis (Table 1) finds only one university offering a core global health degree program in the UAE which is the University of Birmingham Dubai (UoBD) MSc in Global Health System Leadership (13). In addition, and a selection of universities offer global health elective content and cover relevant subject areas such as the Canadian University Dubai BSc in Public Health program and MPH programs at Gulf Medical University and UAE University (Table 1).

**Table 1:** List of global health programs or courses offered in the UAE.

University	Relevant program(s)	Core global health course	Elective global health course
Abu Dhabi University	BSc in public health	No	No
Birmingham University Dubai	MSc in global health system leadership	Yes	No
	Masters in public health	No	No
	MSc in health economics and health policy	Yes (Global health economics)	No
Canadian University Dubai	BSc in public health	Yes	No
Dubai Medical College for Girls	Medical doctor program	Yes (Global and international health)	No
Gulf Medical University	Masters in public health	No	Yes
Hamdan Bin Mohammed Smart University	BSc in health administration	Yes (Introduction to global health)	No
	MSc in public health	No	No
Khalifa University of Science And Technology	PhD in public health	No	No
United Arab Emirated University	Masters in public health	No	Yes
University of Sharjah	Masters in public health	No	No
Zayed University	BSc in public health and nutrition	No	No

It is important to note that these searches may not be fully comprehensive or exhaustive. Relevant global health course content and learning outcomes might be out of view. The range public health programs we identified might well have other connections with global health. Intersections with other subject areas such as public policy and public administration should also be factored in. Mohammed Bin Rashid School of Government’s Master in Public Policy offers global sustainable development alongside health policy and systems thinking.

### A way forward

Our analysis shows that if the UAE is to harness its geopolitical influence and achieve health and education policy priorities, then further development of its education offer is needed.

We are aware that such a perspective and approach is by no means straightforward and indeed requires sensitivity. For example, the predominance of global health degree programs in high-income countries, especially North America and Europe, can be intrinsically connected to the colonial legacy of global health education (14). Explaining the current gap between policy and education provision in the UAE might therefore resonate with calls made elsewhere for greater contextual relevance in order for any global education offer to be enacted.

The experience of developing the MSc in Global Health System Leadership at the University of Birmingham Dubai (UoBD) provides insights into challenges any global health education offer might face (15). This perspective draws on the learning from International Branch Campus (IBC) development to show how curricula can often prioritize the vocational demand for subjects such as business and engineering at the expense of courses in the social sciences and liberal arts, or indeed courses that encourage critical inquiry, experiential learning, and leadership development (5). The interdisciplinary nature of global health education might struggle to take hold within such contexts. High financial costs, admission criteria are tightly tied to English language competencies, possible visa restrictions, and content still likely to draw on predominantly Western-based theories add further limits to an education offer. Governance and policy frameworks also have the potential to provide a limiting factor for global health education. Working in the UAE context requires navigating different norms, rules, and regulations that may challenge academic freedoms.

Despite these various challenges, the UoBD experience of developing a global health education offer shows that by engaging in a spirit of dialogue and reflexivity, progress can be achieved (15). Based on these and other contributions, in the following section we propose the following recommendations in order to move a global health education agenda forward in the UAE.

### Curriculum design

An increase in global health programs and content at both undergraduate and postgraduate levels across UAE universities is needed, either as a compulsory or an elective part of both health science and non-health science curricula. If introducing a full program is not feasible, it is essential to incorporate the principles of global health and notable global health topics such as infectious diseases, climate change, healthcare systems, and health equity within related courses. Most importantly, before the introduction of such programs or courses, further research into the needs and preferences of learners is urgently needed. Based on these expressed needs, delivery options should also be explored with innovation regarding online courses, workshops, study abroad programs, and internships all under consideration. Student-oriented activities such as roundtables for innovative knowledge exchange, hackathons for creative brainstorming, and on-campus surveys to collect qualitative and quantitative data regarding learning preferences.

Knowledge exchange with universities in other countries that provide global health education can provide an additional platform to shape the curriculum. A mapping of global health education activity across the Middle East and the wider MENA region would provide a useful next step in this regard. Learning from best practice with regards to anticolonial approaches to global health education is needed to complement any education agenda moving forward.

### Collaboration and capacity-building

Higher education institutions in the UAE are encouraged to form capacity-building partnerships with local and international global health institutions to build the appropriate capability for global health action. The development of global health institutes and spaces for knowledge exchange can further support any such efforts. A recent partnership agreement between the Canadian University Dubai and University of Oxford to establish The Global Health Network in the MENA region is a step forward (16). The Institute of Public Health at UAE University focus on the global disease burden and public health challenges in the UAE also holds promise for global health research agendas (17).

Existing university associations, such as the Association of Schools of Public Health in the European Region (ASPHER) and the Consortium of Universities for Global Health (CUGH) can provide platforms for further discussion and learning. Encouraging UAE universities to engage in continuous intra-and inter-academic dialog will undoubtedly pave the way for global health education initiatives.

## Conclusion

The UAE’s commitment to a resilient healthcare system and its strategic global partnerships provides an ideal foundation for strengthening global health education. By incorporating comprehensive global health programs and fostering collaboration with international institutions, the UAE can play a significant role in addressing health challenges at both national and global levels. Moving forward, we hope that our Perspective, and its implications, can have the potential to generate further research and understanding about global health education needs in the Middle East, the MENA region, and beyond.

## Data Availability

The original contributions presented in the study are included in the article/supplementary material, further inquiries can be directed to the corresponding author.
